# The COVID-19 pandemic: A multi-regional cross-sectional survey of public knowledge, attitudes, and perceptions

**DOI:** 10.1371/journal.pgph.0000737

**Published:** 2022-07-27

**Authors:** Ismail A. Odetokun, Ahmad I. Al-Mustapha, Hager Elnadi, Deepak Subedi, Oluwaseun A. Ogundijo, Muftau Oyewo

**Affiliations:** 1 Department of Veterinary Public Health and Preventive Medicine, Faculty of Veterinary Medicine, University of Ilorin, Kwara State, Nigeria; 2 Department of Veterinary Public Health and Preventive Medicine, University of Ibadan, Oyo State, Nigeria; 3 Department of Veterinary Services, Kwara State Ministry of Agriculture and Rural Development, Ilorin, Kwara State, Nigeria; 4 Infectious Diseases and One Health, Faculty of Pharmaceutical Sciences, Universite de Tours, Tours, France; 5 Institute of Agriculture and Animal Science, Tribhuvan University, Kirtipur, Nepal; 6 Infectiologie et Santé Publique, Institut National de la Recherche Agronomique, Nouzilly, France; 7 Nigerian Field Epidemiology and Laboratory Training Program, Abuja, Nigeria; PLOS: Public Library of Science, UNITED STATES

## Abstract

With over twelve months since the start of the COVID-19 pandemic, its morbidity and mortality continue to be a critical health threat despite various instituted preventive and control efforts. Information on the multi-regional public perspective of the diseases is limited. Therefore, this study investigated public knowledge, attitudes, and practices towards COVID-19 across Sub-Saharan Africa (SSA), Middle East and North Africa (MENA), and South Asia (SA) regions of the world. In an online pretested questionnaire-based cross-sectional survey, respondents (n = 2738) were recruited using a convenience sampling technique and data obtained were subjected to descriptive and inferential statistics. The majority of respondents had bachelor’s degrees or higher (91.1%) and were aged between 18 and 39 years (88%). Most of the respondents had satisfactory knowledge (73%, 15.4 ± 2.5), attitudes 76.8%, 7.1 ± 1.1, and perceptions (73.4%, 11±2.8). Participants with higher educational levels and scientific backgrounds were 1.71 times (95% CI: 1.44; 2.03; *p <* 0.001) more likely to have a better knowledge of COVID-19. Respondents from the SA region were significantly more likely (OR: 1.4; 95% CI: 1.16, 1.68; *p <* 0.001) to possess satisfactory knowledge of COVID-19. Meanwhile, respondents from the MENA region (OR: 7.81; 95% CI: 6.12, 9.97; *p <* 0.001) have better attitudes and are more optimistic about ending the pandemic than those from the SSA. Despite the satisfactory knowledge, attitudes, and perceptions towards the regional efforts observed, we emphasize continued adherence by the public to the health regulations and safety measures of countries in these regions. There is a need for the low and middle-income countries to improve awareness of COVID-19 preventive practices.

## Introduction

An unusual number of pneumonia cases of unknown origin was described in some patients who visited the Seafood Wholesale Market in Wuhan, Hubei province, China on 31 December 2019 [1, 2]. The causative agent for this unknown disease was identified as a novel type positive-sense RNA genome β-coronavirus on 7 January 2020 [3]. The International Committee on Taxonomy of Viruses (ICTV) named it “severe acute respiratory syndrome coronavirus 2” (SARS-CoV-2, previously referred to as 2019-nCoV). On 11 February 2020, the World Health Organization (WHO) publicized the disease as “COVID-19” [4]. Earlier on 30 January 2020, WHO proclaimed the outbreak as a Public Health Emergency of International Concern (PHEIC) under International Health Regulation (2005) [5]. Later, on 11 March 2020, due to a 13-fold case increase in China, the severity of illness with casualties, and drastic escalation in the number of affected countries, WHO declared the COVID-19 outbreak as a pandemic [6]. As of 31^st^ August 2021, COVID-19 had spread to over 220 countries and territories accounting for about 220 million laboratory-confirmed cases with over 4.5 million mortalities [7]. The extent of the infection and deaths are still uncertain as the disease is still spreading in some countries with different variants been reported.

The major transmission pathway for the disease is from person-to-person through inhalation of aerosols from infected (symptomatic and asymptomatic) individuals [8]. It is highly infectious, and its clinical symptoms include, fever, dry cough, sore throat, dyspnea, diarrhea, fatigue, and myalgia. Reports of critical symptoms have been recorded in certain countries with symptoms ranging from bleeding and coagulation dysfunction, septic shock, unresponsive metabolic acidosis, and acute respiratory distress syndrome [9]. Elderly and patients with pre-existing illnesses (like hypertension, cardiac disease, lung disease, cancer, or diabetes) have been identified as potential risk factors for severe infection and death [10, 11].

Although several COVID-19 vaccines have been produced across the globe [12], the application of preventive measures plays a significant role in disease control and transmission. Regular handwashing with soap and water, constant use of alcohol-based hand sanitizers, a physical distancing of 1–2 feet apart, respiratory hygiene (avoid frequent touching of nose, eyes, and mouth; covering of mouth and nose while coughing or sneezing), use of personal protective wears and the popular stay-at-home cliché (Lockdown) of the year 2020, were all preventive measures adopted in many countries to stop the spread of the disease [13]. Public practice of these preventive measures highly depends upon their knowledge and understanding of the disease.

Earlier in July 2020, there was gradual ease in the lockdown measures in different countries and regions across the world, but the government and several non-governmental organizations need to pay more attention to public awareness and compliance to infection control practices as important means to curtail the continuous transmission of the disease. Public adherence to the preventive and control guideline measures is highly influenced by the knowledge, attitudes, and practices (KAP) of the populace [14] KAP studies of the population in several regions of the world, to assess the extent to which people follow the laid down measures, can be key to further improve communication efforts by the government, public health officials, and clinicians [15], thereby enhancing global health. Therefore, this study investigated public KAP towards COVID19 across the Sub-Saharan Africa (SSA), Middle East and North Africa (MENA), and South Asia (SA) regions of the world.

## Materials and methods

### Study design, study participants, and sampling

A cross-sectional survey of adult respondents (>18 years) from three regions (n = 2738) of the world: SSA (*n* = 1228), MENA (*n* = 441), and SA (*n* = 1069) was carried out from the 9^th^ of April to the 9th of June 2020. These regions were selected based on available classification (UNICEF: https://www.unicef.org/sowc96/groups.htm). The minimum sample size for this study was calculated using the sample size calculator [16] with a possible response distribution of 50%, at 97% confidence level, and an error margin of ±2, which resulted in a sample size of 2566. These three regions were chosen because of the observed increase in the number of persons testing positive for the SARS-CoV-2 virus, per day. Due to the mandatory lockdown measures enforced by most countries of the world, respondents were recruited via online (using a non-systematic approach) social media platforms such as Facebook and WhatsApp with the aid of personal and professional networks and influences. The questionnaire was distributed anonymously online in official languages among respondents from all countries in the three regions. Only countries with more than 10 respondents were included in the analysis. Since the survey was online, the convenience sampling technique was used to identify the participants. More respondents were further recruited using the snowballing technique. However, the study participants were heterogeneous in their nationality, age, gender, and background. In the SSA, the countries included in the study are Burkina Faso, Mali, Nigeria, Senegal, and Rwanda. Bahrain, Egypt, Morocco, and Oman were the countries sampled for MENA while Bangladesh, Nepal, and Pakistan were included as part of the SA countries sampled. Specifications of the World Medical Association Declaration of Helsinki Ethical principles [17] were adhered to during the study, as participation in the survey was without prejudice and informed consent was sought. The confidentiality of the participants’ information was ensured.

### Ethical approval

The Research and Ethics Committee, Kwara State Ministry of Education, Ilorin, Nigeria granted ethical approval (Approval number: DE/PRIM/96/VOL.1/139) for this study. All study participants were provided brief information about the objectives of the survey and their written informed consent was obtained (first question in the survey instrument). Only those who gave written consent were included in the study and participants could decline participation and opt-out at any time.

### Questionnaire design

A self-administered structured questionnaire previously designed and used in a preliminary KAP survey on COVID-19 in Egypt and Nigeria [18] was utilized in this survey. Consent to use this survey tool was sought and approval granted. This questionnaire was primarily designed in English language (with the option of auto-translation to other languages by Google Translate). These translations have already been approved in Arabic and French by language experts who analyzed the linguistic contents during the translation-back translation exercise. To assess the content validity, clarity, ease of response, scope, and face validity of the questions for suitability in a multi-regional setting, three independent reviewers were selected to validate the questionnaire. Furthermore, to check for technical glitches, the aptness of the survey tool, and typographical errors, a pre-test survey was performed on 20 volunteers drawn from the surveyed regions. Responses obtained were not incorporated in the final analysis of the data.

The demographic characteristics of the respondents (age, gender, education, educational background, and region) were determined in the first part (Section A in S1 File) of the questionnaire. The knowledge about the COVID-19 pandemic, attitudes concerning prevention of the disease, and perceptions on institutionalized efforts was asked in Sections B to E in S1 File. The knowledge questions focused on participants’ understanding of the disease, its transmissibility, signs, and symptoms in the infected individuals, the incubation period of the disease, and how to limit the infection. Questions harvesting information about respondents’ attitudes to the use of protective and preventive practices such as standard handwashing, face mask use, and social distancing/self-isolation were asked in Section C in S1 File. Lastly, Sections D and E in S1 File comprised questions evaluating the participants’ perceptions of global and community response efforts geared towards the control of the pandemic and preventing against future outbreaks (Sile1).

### Data analysis

The Microsoft Excel 2019 was utilized to summarize the data collected and subjected to further statistical analysis using the Open-Source Epidemiologic Statistics for Public Health (OpenEpi), v.3.01 (updated 2013/04/06), and the Statistical Package for the Social Sciences (SPSS) software, v.22. The demographic information of participants (independent variables) was summarized using univariate analysis (frequency and proportions). To subject the responses on knowledge, attitudes, and perceptions to inferential statistics, first a numeric scoring model was employed [19]. The knowledge, attitudes, and perceptions levels of respondents were treated as the outcome (dependent) variables. For ease of analysis, these dependent variables were further classified as satisfactory or unsatisfactory (binary variables) centered on computed cut-off points. Scores greater than the mean scores for knowledge (15.4±2.5), attitudes (7.1±1.1), and perceptions (11.0±2.8) obtained by the respondents were considered satisfactory and vice versa. To test for association between the demographic characteristics of respondents and the three outcome variables, the Chi-square (χ^2^) test was utilized. All analyses were carried out at a confidence interval of 95% and variables with significant values (*p*<0.05) at the level of test for association were subjected to binary logistic regression analysis.

## Results

### Respondent demographics

A total of 2738 participants were recruited from 12 countries from the three regions. Most of the respondents (91.1%) had a bachelor’s degree or more and 88% (*n* = 2409) were aged between 18 and 39 years. The responses were made up of mostly respondents (59.1%, *n* = 1618/2738) with non-scientific backgrounds (Table 1).

**Table 1:** Demographics of respondents from Sub-Saharan Africa, the Middle east, and North Africa and South Asia (n = 2738).

Variable	Number of respondents (%)
***Age (years)***	
18–29	1744 (63.7)
30–39	665 (24.3)
40–49	237 (8.7)
50–59	67 (2.4)
>59	25 (0.9)
***Gender***	
Male	1411 (51.5)
Female	1315 (48.1)
Prefer not to say	6 (0.4)
***Education***	
No formal education	90 (3.3)
High School	155 (5.7)
College (Bachelor)	1845 (67.4)
Masters	533 (19.5)
Ph.D.	115 (4.2)
***Background***	
Non-Scientific/Non-Medical	1618 (59.1)
Scientific/Medical	1120 (40.9)
***Regions***	
Sub-Saharan Africa	1228 (44.9)
The Middle East and North Africa	441 (16.1)
South Asia	1069 (39.0)

### Participants’ knowledge of the COVID-19 pandemic

The majority of the participants had satisfactory knowledge, attitudes and perceptions towards COVID-19 and the community and global efforts aimed at controlling the pandemic, with mean scores of 15.4 ± 2.5 (72.9%, *n* = 1997/2738), 7.1 ± 1.1 (76.8%, *n* = 2104/2738) and 11.0 ± 2.8 (73.4%, *n* = 2011/2738), respectively (Table 2). Table 3 showed that 99.3% of the respondents (*n* = 2719/2738) were aware of the COVID-19 pandemic. However, some (29.5%, *n* = 808/2738) thought it was the same as the common flu. The satisfactory knowledge was evident in the fact that most of the respondents knew that there could be asymptomatic infections (81.4%, *n* = 2230/2738), that the incubation period is between 1–14 days or 2–21 days (97.9%, *n* = 2680/2738), and also knew that all age groups could be infected with COVID-19 (98.5%, *n* = 2697/2738). In the same vein, most of the respondents knew the importance of handwashing in preventing transmission of the virus (99.6%, *n* = 2728/2738), and the recommended duration for hand hygiene (92%, *n* = 2520/2738).

**Table 2:** Description of obtainable scores (outcomes) by participants (n = 2738).

Outcome variables	Maximum obtainable scores	Scores obtained by Participants		Mean ± SD	Satisfactory* n (%)	Unsatisfactory n (%)
		Minimum score	Maximum score			
Knowledge	20	3	19	15.4 ± 2.5	1997 (72.9)	741 (27.1)
Attitudes	9	2	17	7.1 ± 1.1	2104 (76.8)	634 (23.2)
Perceptions	17	1	16	11.0 ± 2.8	2011 (73.4)	727 (26.6)

*—Satisfactory scores are calculated as scores obtained by the participants which are greater than the average scores (cut-off points) for knowledge (15.4±2.5), attitudes (7.1±1.1), and perceptions (11.0±2.8).

**Table 3:** Knowledge of respondents about the COVID-19 pandemic in the three regions.

Variables	No. of respondents (%)
***Have you heard of the COVID-19***	
No	19 (0.7)
Yes	2719 (99.3)
***Is the COVID-19 virus the same as the common cold (flu) virus*?**	
No	808 (29.5)
Yes	1930 (70.5)
*Is it possible for a COVID-19 positive person to show no symptoms*?	
No	508 (18.6)
Yes	2230 (81.4)
* ***How long does it take from contracting the disease to showing symptoms*?**	
1–14 days	2409 (87.9)
2–21 days	271 (9.9)
1–3 months	25 (0.9)
I don’t know	25 (0.9)
* ***Who can get infected with COVID-19*?**	
Anyone can be infected	2697 (98.5)
People with chronic diseases only	22 (0.8)
Old people only	15 (0.5)
Teenagers and children only	2 (0.1)
Young adults only	2 (0.1)
* ***Which is a symptom for COVID-19*?**	
High fever	2575 (94.1)
Dry cough	2450 (89.5)
Difficulty in breathing	2616 (95.5)
Muscle pain	968 (35.4)
Fatigue	1389 (50.7)
Runny nose	1192 (43.5)
Bleeding	83 (3.0)
Hair loss	29 (1.1)
I don’t know	5 (0.2)
* ***How does the virus spread*?**	
Air droplets (from patient sneezing/coughing)	2511 (91.7)
Contact with contaminated surfaces	2222 (81.2)
Close contact with people who have the virus	2461 (89.9)
Mosquitos/flies bites	30 (1.1)
* ***What can kill the virus*?**	
Alcohol-based sanitizers	2492 (91.0)
Soap/detergents	2173 (79.4)
Clean surfaces with diluted chlorine	1411 (51.5)
Water alone	88 (3.2)
All of the above	108 (3.9)
I don’t know	66 (2.4)
***Is handwash important*?**	
No	10 (0.4)
Yes	2728 (99.6)

*—Multiple choice question

The Demographic characteristics (gender, educational level, and background, and region) had a significant association with the knowledge levels of the respondents (Table 4). However, with binary logistic regression analysis, participants with higher educational levels and of scientific backgrounds were 1.71 × (95% Confidence interval (CI): 1.44; 2.03; *p*<0.001) more likely to have a better knowledge of COVID-19. Participants from South Asia were significantly more likely (Odds ratio (OR): 1.4; 95% CI: 1.16, 1.68; *p*<0.001) to possess satisfactory knowledge of COVID-19 (Table 4).

**Table 4:** Binary logistic regression analysis of demographic factors affecting the knowledge of respondents about COVID-19 pandemic from the three regions.

Demographic factors	Satisfactory (%)	Unsatisfactory (%)	P—value (χ2)	Odds ratio	95% Confidence interval	P—value
***Age***						
18–29	1244 (71.3)	500 (28.7)	0.05	-		
30–39	514 (77.3)	151 (22.7)		-	-	-
40–49	170 (71.7)	67 (28.3)		-	-	-
50–59	49 (73.1)	18 (26.9)		-	-	-
>59	20 (80.0)	5 (20.0)		-	-	-
***Gender***						
Male	1008 (71.4)	403 (28.6)	0.03*	-		
Female	983 (74.8)	332 (25.2)		0.84	0.71, 1.00	0.057
^λ^Prefer not to say	6 (50.0)	6 (50.0)				
***Education***						
No formal education	59 (65.6)	31 (34.4)	< 0.01*	-		
High School	87 (56.1)	68 (43.9)		1.49	0.87, 2.55	0.188
College (Bachelor)	1344 (72.7)	501 (27.3)		0.71	0.45, 1.11	0.168
Masters	415 (77.9)	118 (22.1)		0.54	0.33, 0.87	0.019*
Ph.D.	92 (80.0)	23 (20.0)		0.48	0.25, 0.89	0.030*
***Background***						
Non—Scientific/Non–Medical	1251 (77.3)	367 (22.7)	<0.001*	-		
Scientific/Medical	746 (66.6)	374 (33.4)		1.71	1.44, 2.03	<0.001*
***Region***						
Sub-Saharan Africa	930 (75.7)	298 (24.3)	0.001*	-		
Middle east and North Africa	328 (74.4)	113 (25.6)		1.08	0.84, 1.38	0.613
South Asia	739 (69.1)	330 (30.9)		1.39	1.16, 1.68	<0.001*

**χ2** –chi-square

**λ** - excluded from the binary logistic regression analysis

*—significant at p < 0.05.

#### Participants’ attitudes towards the COVID-19 pandemic

Most of the respondents to this survey demonstrated a positive attitude towards preventive measures instituted against the COVID-19 outbreaks such as the use of proper hygiene (96.1%, *n* = 2630/2738), and self-isolation/social distancing (83.2%, *n* = 2277/2738), and the use of face masks/gloves (88.3%, *n* = 2418/2738) (Table 5). Similarly, most respondents (98.9%, *n* = 2709/2738) followed the governments’ regulations stipulated in their regions. All the demographic characteristics have a significant association with the attitudes of the respondents (Table 6). Respondents who were between 40–49 years (OR: 2.61; 95% CI: 1.96, 3.49; *p*<0.001) and having Master education (OR: 0.44; 95% CI: 0.27, 0.71; *p*<0.002) were predictors of positive attitudes towards the preventive measures established to curb the spread of the virus. The respondents from the MENA region (OR: 7.81; 95% CI: 6.12, 9.97; *p*<0.001) were more likely to have better attitudes and more optimistic about ending the pandemic than those from Sub-Saharan Africa (Table 6).

**Table 5:** Descriptive statistics of respondents’ attitudes to the COVID-19 pandemic in the three regions.

Variables	Number of respondents (%)
*Which of these can be protective against COVID-19?	
Proper hygiene (handwash/cover mouth and nose during coughing or sneezing)	2630 (96.1)
Self- Isolation/ Social distancing	2277 (83.2)
Face masks/gloves	2418 (88.3)
Garlic, Onions, and Ginger	705 (25.7)
Antibiotics	274 (10.0)
Others	38 (1.4)
* ***Who should wear a facemask*?**	
Everyone	1775 (64.8)
Only sick people	831 (30.4)
People in contact with the ill	1140 (41.6)
Health workers	1139 (41.6)
***Do you think social distancing/self-isolation is an effective measure to reduce the spread of COVID-19*?**	
Yes	2632 (96.1)
No	35 (1.3)
Maybe	71 (2.6)
***What is the ideal distance between two people during social distancing*?**	
No distance	3 (0.1)
Less than one meter	52 (1.9)
1–2 meters	1850 (67.6)
3–5 meters	591 (21.6)
Greater than 5 meters	164 (5.9)
I don’t know	48 (1.8)
No response	30 (1.1)
***Do you follow the recommendation of your Health Ministry or government*?**	
No	29 (1.1)
Yes	2709 (98.9)
***Do you think that your government is doing enough to stop the pandemic*?**	
No	1669 (61.0)
Yes	1069 (39.0)
***Do you agree with the obligatory lockdown measures your country is taking*?**	
No	530 (19.4)
Yes	2208 (80.6)

*—Multiple choice question

**Table 6:** Binary logistic regression analysis of demographic factors affecting respondents’ attitudes about the COVID-19 pandemic from the three regions.

Demographic factors	Satisfactory (%)	Unsatisfactory (%)	P—value (χ2)	Odds ratio	95% Confidence interval	P—value
***Age***						
18–29	1413 (81.0)	331 (19.0)	<0.01*	-		
30–39	475 (71.4)	190 (28.6)		1.71	1.39, 2.09	<0.001*
40–49	147 (62.0)	90 (38.0)		2.61	1.96, 3.49	<0.001*
50–59	53 (79.1)	14 (20.9)		1.13	0.62, 2.06	0.793
>59	16 (64.0)	9 (36)		2.40	1.05, 5.48	0.072
***Gender***						
Male	1128 (79.9)	283 (20.1)	<0.001*	-		
Female	969 (73.7)	346 (26.3)		1.42	1.19, 1.70	<0.001*
^λ^Prefer not to say	7 (58.3)	5 (41.7)				
***Education***						
No formal education	59 (65.6)	31 (34.4)	0.001*	-		
High School	112 (72.3)	43 (27.7)		0.73	0.42, 1.28	0.338
College (Bachelor)	1422 (77.1)	423 (22.9)		0.57	0.36, 0.89	0.021*
Masters	433 (81.2)	100 (18.8)		0.44	0.27, 0.71	0.002*
Ph.D.	78 (67.8)	37 (32.2)		0.90	0.50, 1.62	0.845
***Background***						
Non—Scientific/Non—Medical	1315 (81.3)	303 (18.7)	<0.001*	-		
Scientific/Medical	789 (70.4)	331 (29.6)		1.81	1.52, 2.17	<0.001*
***Region***						
Sub-Saharan Africa	1028 (83.7)	200 (16.3)	<0.001*	-		
Middle East and North Africa	175 (43.6)	266 (56.4)		7.81	6.12, 9.97	<0.001*
South Asia	901 (84.3)	168 (15.7)		0.96	0.77, 1.19	0.753

**χ2**—chi square

**λ** - excluded from the binary logistic regression analysis

*—significant at p < 0.05

#### Perceptions of the partcicipants about the COVID-19 pandemic

Furthermore, most of the respondents (61%, *n* = 1669/2738) do not believe that their government is doing enough to control the transmission of the virus. Some of the participants (19.4%, *n* = 530/2738) do not agree with the obligatory lockdown instituted by their local authorities (Table 7). Only 64.5% (*n* = 1767/2738) of the study participants believed that the occurrence of future pandemics could be forestalled. Of the demographic variables, only educational level and background were associated with the participant’s perceptions of the local and global efforts to controlling the pandemic (Table 8). Respondents with scientific backgrounds (OR: 1.43; 95% CI: 1.21, 1.69; *p*<0.001) were more likely to have a more satisfactory perception of the community and global concerted efforts aimed at controlling the virus (Table 8).

**Table 7:** Descriptive statistics of respondents’ perceptions of community and global responses associated with the prevention of the pandemic.

Variable	Number of respondents (%)
***Do you think that your government is doing enough to stop the pandemic*?**	
No	1669 (61.0)
Yes	1069 (39.0)
***Do you agree with the obligatory lockdown measurements your country is taking*?**	
No	530 (19.4)
Yes	2208 (80.6)
* ***What do you think we can do as a community to reduce the spread of COVID-19*?**	
Follow/respect the health recommendations of my country	2538 (92.7)
Eat healthy/ Practice sports	1663 (60.7)
Social distancing/Avoid the crowd	2466 (90.1)
Volunteer to support whenever possible	1368 (49.9)
Avoid handshakes and face kissing	2216 (80.9)
Attending religious gatherings	53 (1.9)
I don’t know	25 (0.9)
No response	96 (3.5)
***Do you think we can prevent such a global pandemic in the future*?**	
No	971 (35.5)
Yes	1767 (64.5)
* ***Which of these practices can prevent/help against the occurrence of such a global pandemic in the future*?**	
Establish early alerts and global warning systems for infectious diseases	2106 (76.9)
Intensify research on preventive measures such as vaccines/diagnosis	2024 (73.9)
Raise public awareness of proper hygiene/healthy habits	2249 (82.1)
Improve surveillance in the human and animal health sectors	1895 (69.2)
Collaboration between environmental, animal, and human health workers	1713 (62.6)
Reduce international travels	990 (36.2)
Prioritize human life/health welfare over the animal or environmental ones	699 (25.5)
Intensify research on preventive measures such as vaccines/diagnoses	59 (2.2)
***Are you willing to read and share with others information on COVID-19*?**	
No	268 (9.8)
Yes	2470 (90.2)

*—Multiple choice question

**Table 8:** Binary logistic regression analysis of demographic factors affecting respondents’ perceptions about the COVID-19 pandemic from the three regions.

Demographic factors	Satisfactory (%)	Unsatisfactory (%)	P-value (χ2)	Odds ratio	95% Confidence interval	P-value
***Age***						
18–29	1274 (73.1)	470 (26.9)	0.973	-		
30–39	491 (73.8)	174 (26.2)		-	-	-
40–49	177 (74.7)	60 (25.3)		-	-	-
50–59	50 (74.6)	17 (25.4)		-	-	-
>59	19 (76.0)	6 (24.0)		-	-	-
***Gender***						
Male	1026 (72.7)	385 (27.3)	0.108	-		
Female	979 (74.4)	336 (25.6)		-	-	-
Prefer not to say	6 (50.0)	6 (50.0)		-	-	-
***Education***						
No formal education	64 (71.1)	26 (28.9)	0.009*	-		
High School	96 (61.3)	59 (38.7)		1.51	0.86, 2.65	0.187
College (Bachelor)	1358 (73.6)	487 (26.4)		0.88	0.55, 1.41	0.678
Masters	408 (76.5)	125 (23.5)		0.75	0.46, 1.24	0.326
Ph.D.	85 (73.9)	30 (26.1)		0.87	0.469, 1.61	0.771
***Background***						
Non—Scientific/Non–Medical	1235 (76.3)	383 (23.7)	<0.001*	-		
Scientific/Medical	776 (69.3)	344 (30.7)		1.43	1.21, 1.69	<0.001*
***Region***						
Sub-Saharan Africa	910 (74.1)	318 (25.9)	0.744	-		
Middle east and North Africa	324 (73.5)	117 (26.5)		-	-	-
South Asia	777 (72.7)	292 (27.3)		-	-	-

χ2—Chi square

**λ** - excluded from the multivariable logistic regression analysis

*—significant at p < 0.05.

## Discussion

Currently, the number of confirmed cases due to COVID-19 is over 230 million, which has led to over 4.7 million reported deaths worldwide [20]. For effective control of the pandemic, citizens need to know the basic facts about the disease and possess the right attitudes. This will enhance general intervention and policy decisions on the control of the pandemic.

Most respondents to this survey demonstrated a satisfactory knowledge about the Coronavirus disease. They were also aware of the preventive measures and the need for the control measures being put in place. This high awareness rate and knowledge level can be attributed to the fact that most of the participants (91.1%) had a good educational background. The series of awareness campaigns, social media posts, and updates could have further contributed to the satisfactory knowledge of the respondents. This, however, cannot be generalized for the entire population of the regions as this survey could mainly assess mostly young (18–39 years old) and literate internet users. Similarly, studies conducted in China, and Iran showed higher knowledge levels of the respondents with an overall knowledge score exceeding 90% [14, 21]. The regional differences in the knowledge and attitudes toward COVID-19 and its preventive measures could mainly be due to the different risk communication and mass advocacy strategies employed by several countries of the surveyed regions as well as its enforcement of the preventive measures.

There was a significant association between age, gender, level of education, background, and the region on the overall knowledge score. This is expected as knowledge is usually a correlate of these demographic factors. For instance, in some earlier reports–similar to our findings–from India, China, Egypt, and Nigeria, those with some levels of education and are young exhibited better knowledge of the COVID-19 pandemic [14, 22–25]. The almost seamless access to social media via the internet appears to be a major contributory factor to this. Social media platforms have been integral in information dissemination during the pandemic. It has also been cardinal in assisting people all over the world to adapt to lockdown protocols. The availability of a plethora of online courses has helped in raising the knowledge level of the study participants and has helped them gained new skills [26].

Although most countries in the three regions are under full lockdown, and the mean attitude score of 7.1±1.1 showed that the citizens are optimistic for a bright post-pandemic future. This is evident by the majority of respondents who showed a confident attitude regarding preventive measures instituted in their countries to counter the spread of the pandemic. This positive attitude could be attributed to a good knowledge of the disease, and the fact that some countries are beginning to ease their lockdown measures. Most of the study participants valued and practiced social distancing (96.1%) and followed the stipulations established by their health authorities (98.9%). However, the attitudes toward the pandemic was significantly different within regions. This difference could be a result of the harsh economic privation experienced by the citizens of certain regions (mostly the low- and middle-income countries) as some countries have a high poverty index; and a vast population of workers whose survival is based on their daily earnings or wages. Inadequate government palliative policies for the populace are capable of affecting the overall success of COVID-19 prevention and control [27]. To support this, 61% of the respondents to our survey believe that their government’s efforts are inadequate to control the current Coronavirus pandemic. This indicates that governments of respective countries across the sampled regions need to improve their investment in the control of the pandemic and gain the trust of their citizens as their opinions would influence the success of any government interventions during the control and prevention efforts.

In this study, the percentage (96%) of the survey participants having a positive perception of self-isolation being essential and effective is high, which made them avoid persons/places positive for COVID-19. This might be correlated to the lower number of confirmed cases recorded and noticed initially across these regions. However, this is changing as governments across the world are beginning to relax the lockdown protocols to avoid economic collapse. Though it is essential to maintain a safe balance between safeguarding public health and avoiding economic collapse, more emphasis should be placed on social distancing, personal and community hygiene, and enforcement of strict use of personal protective equipment such as the facemask, especially in public spheres.

Most respondents have a good perception of the COVID-19 pandemic, as 73.4% of the respondents had a mean score of 11.0±2.8. The majority of the respondents (64.5%) believed that collectively we can prevent future outbreaks of similar pandemics. Some of the respondents (46%) complemented the efforts of the WHO in the global control of the pandemic and in providing health leadership, technical guidance, and coordinating the COVID-19 research for a possible vaccine. Besides, the support for the WHO’s efforts might be attributed to the timely updates on the disease burden, press conferences, dissemination of trusted information, travel advice, and strengthening of the health services [28] especially in Sub-Saharan Africa and South Asia.

Although the majority of the respondents had non-scientific/non-medical backgrounds, they acknowledged the need for an increased multi-sectorial One-health collaboration of human, animal, and environmental health professionals to prevent spillover of pathogens from animals to humans. Also, respondents believed that establishing early alerts and global warning systems for infectious diseases will help reduce the impact of any future pandemic. Essentially, more educational and purposeful enlightenment campaigns are needed to increase awareness and public understanding about the diseases across regions, especially among low internet users in the populace. Awareness campaigns are an important component of infection prevention and control [29] and have been used to prevent COVID-19 in Afghanistan [30].

This study is not without some limitations. For instance, this study was skewed in favor of high internet users while low responses were obtained from several persons with limited access to the internet across the three surveyed regions. This, coupled with the mandatory lockdown restricted the number of consenting respondents to this survey across the three regions to 2,738. Furthermore, the majority of the respondents were young, and between 18 to 39 years. This is largely due to heightened interest in social media observed among these young populations. However, we believe that the public knowledge, attitudes, and perceptions toward the COVID-19 pandemic observed among these high internet users would not be different significantly from others as both the high and limited internet users cohabit together within the same society. More so, this is the first survey to our knowledge to present a cross-regional insight into the KAP of the current pandemic using a convenience sampling technique in a cross-sectional survey. Therefore, it provides rapid insight into the current KAP concerning the pandemic circulating among the public. Research based on the use of a robust and systematic sampling protocol is needed to further improve the generalizability and external validity of the findings. Also, future research should target more regions on a global scale to enhance policy and decision-making on a global level to ensure that the pandemic is halted.

## Conclusion

Respondents from the three regions were aware of the pandemic, had good knowledge, satisfactory attitudes, and perceptions toward the global response. However, we recommend strict individual and public adherence to the health guides and safety measures of the countries within these regions, most importantly, as the ease in lockdown begins to trend. Greater emphasis should be placed on physical distancing, personal and community hygiene, and enforcement of strict use of personal protective equipment such as the facemask, especially in public places. There is a need for regions to improve upon current preventive actions. Respective governments, especially in the low- and middle-income countries should be engaged in more educational and purposeful enlightenment campaigns from the grassroots to urban centers to increase awareness about infection and control practices needed to adequately control the pandemic.

## Supporting information

S1 File Knowledge and Attitudes towards the 2019 coronavirus pandemic: A cross-sectional social sciences survey. (DOCX)

## References

[1] World Health Organization (WHO). Pneumonia of unknown cause–China [Internet]. Who.int. 2021 (Accessed 28 August 2021). Available from: https://www.who.int/emergencies/disease-outbreak-news/item/2020-DON229

[2] Lu H, Stratton CW, Tang YW. Outbreak of pneumonia of unknown etiology in Wuhan China: The mystery and the miracle, J Med Virol. 2020. doi: 10.1002/jmv.25678 31950516 PMC7166628

[3] Gorbalenya AE, Baker SC, Baric RS, de Groot RJ, Drosten C, Gulyaeva AA, et al. The species severe acute respiratory syndrome-related coronavirus: Classifying 2019-nCoV and naming it SARS-CoV-2. Nat Microbiol. 2020. doi: 10.1038/s41564-020-0695-z 32123347 PMC7095448

[4] World Health Organization (WHO). Naming the coronavirus disease (COVID-19) and the virus that causes it. 2020a. (Accessed 12 July 2020). Available from: https://www.who.int/emergencies/diseases/novel-coronavirus2019/technical-guidance/naming-the-coronavirus-disease-(COVID-2019)-and-the virus-that-causes-it

[5] World Health Organization (WHO). The 2019-nCoV outbreak is an emergency of international concern. 2020b. http://www.euro.who.int/en/healthtopics/emergencies/pages/news/news/2020/01/2019-ncov-outbreak-is-an-emergency-of-international-concern (Accessed Feb 16, 2020).

[6] Cucinotta D, Vanelli M. WHO declares COVID-19 a pandemic. Acta Biomed., 2020;91(1): 157–160. doi: 10.23750/abm.v91i1.9397 32191675 PMC7569573

[7] Worldometer. COVID-19 Coronavirus Pandemic. Dover, Delaware: Worldometers.info. (2020). Available online: https://www.worldometers.info/coronavirus/?utm_campaign=homeAdUOA?Si (Accessed 31 August 2021).

[8] Huang C, Wang Y, Li X, Ren L, Zhao J, Hu Y, et al. Clinical features of patients infected with 2019 novel coronavirus in Wuhan, China. Lancet. 2020;395(10223):497–506. doi: 10.1016/S0140-6736(20)30183-5 31986264 PMC7159299

[9] Chen N, Zhou M, Dong X, Qu J, Gong F, Han Y, et al. Epidemiological and clinical characteristics of 99 cases of 2019 novel coronavirus pneumonia in Wuhan, China: a descriptive study. Lancet. 2020;395(10223):507–513. doi: 10.1016/S0140-6736(20)30211-7 32007143 PMC7135076

[10] Shi H, Han X, Jiang N, Cao Y, Alwalid O, Gu J, et al. Radiological findings from 81 patients with COVID-19 pneumonia in Wuhan, China: a descriptive study. Lancet Infect Dis. 2020;20:425–34. doi: 10.1016/S1473-3099(20)30086-4 32105637 PMC7159053

[11] Tian S, Hu N, Lou J, Chen K, Kang X, Xiang Z, et al. Characteristics of COVID-19 infection in Beijing. J Infect. 2020;80:401–6. doi: 10.1016/j.jinf.2020.02.018 32112886 PMC7102527

[12] Sahin AR, Erdogan A, Agaoglu PM, Dineri Y, Cakirci AY, Senel ME, et al. 2019 novel coronavirus (COVID-19) outbreak: a review of the current literature. EJMO. 2020;4:1–7. doi: 10.14744/ejmo.2020.12220

[13] World Health Organisation (WHO). Coronavirus Disease (COVID-19) Advice for the Public. 2020c. Available online at: www.who.int/emergencies/diseases/novelcoronavirus-2019/advice-for-public (Accessed 30 May 2020).

[14] Zhong BL, Luo W, Li HM, Zhang QQ, Liu XG, Li WT, et al. Knowledge, attitudes, and practices towards COVID-19 among Chinese residents during the rapid rise period of the COVID-19 outbreak: a quick online cross-sectional survey. International journal of biological sciences. 2020;16(10):1745–1752. doi: 10.7150/ijbs.45221 32226294 PMC7098034

[15] Balkhy HH, Abolfotouh MA, Al-Hathlool RH, Al-Jumah MA. Awareness, attitudes, and practices related to the swine influenza pandemic among the Saudi public. BMC infectious diseases. 2010; 10(1):42. 10.1186/1471-2334-10-42 20187976 PMC2844401

[16] RAOSOFT. Sample Size Calculator 2020. Available online: http://www.raosoft.com/samplesize.html (Accessed 25 March, 2020).

[17] World Medical Association (WMA). World Medical Association Declaration of Helsinki ethical principles for medical research involving human subjects. JAMA, 2013; 310:2191–2194. doi: 10.1001/jama.2013.281053 24141714

[18] Elnadi H, Odetokun IA, Obasanjo B, Ahmed Z, Ochulor O, Al-Mustapha AI. Knowledge, attitude, and perceptions towards the 2019 Coronavirus Pandemic: A bi-national survey in Africa. 10.1101/2020.05.27.20113951 PMC7390376 32726340

[19] Odetokun I, Akpabio U, Alhaji N, Biobaku K, Oloso N, Ghali-Mohammed I et al. Knowledge of antimicrobial resistance among veterinary students and their personal antibiotic use practices: A National cross-sectional survey. Antibiotics. 2019;8(4):243. doi: 10.3390/antibiotics8040243 31795341 PMC6963658

[20] John Hopkins University Coronavirus Dashboard. COVID-19 Map [Internet]. Johns Hopkins Coronavirus Resource Center. 2020 [Cited 23 September 2021]. Available from: https://coronavirus.jhu.edu/map.html (Accessed on 7 July 2020).

[21] Erfani A, Shahriarirad R, Ranjbar K, Mirahmadizadeh A, Moghadami M. Knowledge, Attitude and Practice toward the Novel Coronavirus (COVID-19) Outbreak: A Population—Based Survey in Iran. [Preprint]. Bull World Health Organ. E—pub: 30 March 2020. 10.2471/BLT.20.256651

[22] Abdelhafiz AS, Mohammed Z, Ibrahim ME, et al. Knowledge, Perceptions, and Attitude of Egyptians Towards the Novel Coronavirus Disease (COVID-19) [published online ahead of print, 2020 Apr 21]. J Community Health. 2020;1‐10. doi: 10.1007/s10900-020-00827-7 32318986 PMC7173684

[23] Roy D, Tripathy S, Kar S, Sharma N, Verma S, Kaushal V. Study of knowledge, attitude, anxiety & perceived mental healthcare need in Indian population during COVID-19 pandemic. Asian Journal of Psychiatry. 2020;51:102083. doi: 10.1016/j.ajp.2020.102083 32283510 PMC7139237

[24] Reuben RC, Danladi MMA, Saleh DA et al. Knowledge, Attitudes and Practices Towards COVID-19: An Epidemiological Survey in North-Central Nigeria. J Community Health 2020. 10.1007/s10900-020-00881-1 PMC7338341 32638198

[25] Al-Hanawi MK, Angawi K, Alshareef N, et al. Knowledge, Attitude and Practice Toward COVID-19 Among the Public in the Kingdom of Saudi Arabia: A Cross-Sectional Study. Front Public Health. 2020;8:217. doi: 10.3389/fpubh.2020.00217 32574300 PMC7266869

[26] Rugarabamu S, Byanaku A. Knowledge, attitudes, and practices (KAP) towards COVID-19: A quick online cross-sectional survey among Tanzanian residents. 2020. 10.1101/2020.04.26.20080820.

[27] Solberg L.B. The Benefits of Online Health Communities. American Medical Association Journal of Ethics. 2014; 164: 270–274. doi: 10.1001/virtualmentor.2014.16.04.stas1-1404 24735575

[28] Costantini M, Sleeman KE, Peruselli C, Higginson I.J. Response and role of palliative care during the COVID-19 pandemic: A national telephone survey of hospices in Italy. Palliative Medicine 2020;34(7):889–895. doi: 10.1177/0269216320920780 32348711 PMC7218350

[29] World Health organization (WHO). Campaigns. 2020d. Available from: https://www.who.int/infection-prevention/campaigns/en/ (Accessed 23 June 2020)

[30] World Bank. Awareness Campaigns Help Prevent Against COVID-19 in Afghanistan. 2020. https://www.worldbank.org/en/news/feature/2020/06/28/awareness-campaigns-help-prevent-against-covid-19-in-afghanistan (Accessed 23 June 2020)

