# Supplementary material for: The COVID-19 pandemic: A multi-regional cross-sectional survey of public knowledge, attitudes, and perceptions
Source: PLOS Glob Public Health. 2022 Jul 27;2(7):e0000737. doi: 10.1371/journal.pgph.0000737 (PMC10022257; doi:10.1371/journal.pgph.0000737)
Supplement: S1 File — (DOCX) [file pgph.0000737.s001.docx]

**Knowledge and Attitudes towards the 2019 Coronavirus Pandemic: A Cross-Sectional Social Sciences Survey**

This survey was designed to generate data that describes gaps in the knowledge and perceptions of respondents; which will be used to improve our infographic meant to contribute to global control of the pandemic. Information will be kept confidential and for research purposes only. Kindly note that this survey is voluntary and you can withdraw at any point. NB: COVID-19 is the disease caused by the SARS-CoV-2 virus. Correspondence email: ai.almustapha42@gmail.com

**CONSENT**

Would you like to participate in this survey?

YES (Kindly append your signature)

NO

**SECTION A: DEMOGRAPHICS**

Age (Years):

- 18-29
- 30-39
- 40-49
- 50-59
- 60-69
- >69 years

Sex:

- Male
- Female
- Prefer not to say

Level of Education:

No formal education

High School

College

Master

PhD

Background

- Scientist/Medical
- Non-Scientific/Non-medical

Nationality:

**SECTION B: KNOWLEDGE OF CORONAVIRUS**

1. Have you heard of the COVID-19? Yes No
2. Where did you hear of it?

1)TV 2) Internet (Social media) 3) Newspapers 4) Friends/family 5) Others

1. Is the COVID-19 virus the same as the common cold virus?

Yes No I don’t Know

1. Is it possible for a COVID-19 positive person to show no symptoms?

Yes No

1. How long does it take from contracting the disease to showing symptoms?

1) 2-12 days

2) 1-3 months

3) 1-4 days

4) I don’t know

1. Who can get infected with COVID-19?

- Old people only
- Young adults only
- Anyone can be infected
- Teenagers and children only
- People with chronic diseases only

1. Which is a symptom for COVID-19? (Choose all that applies)

- High Fever
- Runny nose
- Dry cough
- Breathing difficulty
- Hair loss
- Muscle pain
- Fatigue
- Bleeding

1. How does the virus spread?
   - - Air droplets
     - Mosquitoes/flies bite
     - Contact with contaminated surfaces
     - Close contact with people who have the virus
2. What can kill the virus?

Clean surfaces with diluted chlorine

Alcohol based sanitizers

Soap/detergents

Water alone

I don’t know

1. Is handwash important? Yes No
2. If yes in 10 above, How long should you wash your hands to kill the virus?

- Less than 20 seconds
- 20 seconds to 1 minute
- 1 minute to 3 minutes
- 3 minutes to 5 minutes
- > 5minutes
- I don’t know

**SECTION C: ATTITUDE TOWARDS PREVENTIVE MEASURES**

1. Which of these can be protective against COVID-19

proper hygiene (handwash/cover mouth and nose during coughing or sneezing)

- Close contact with people/crowd
- Face masks/gloves
- Antibiotics
- Ginger, Onions and Garlic
- Others

1. Who should wear a face mask?

- Everyone
- Only sick people
- People in contact with the ill
- Health workers

1. Do you think social distancing/self-isolation is an effective measure to reduce the spread of the SARS-COV-2(COVID-19)?

Yes No I don’t know

1. What is the ideal distance between two people during social distancing?

- No distance
- Less than 1 meter
- 1-2 meters
- 3-5 meters
- >5 meters
- I don’t know

1. Do you follow the recommendations of your Health ministry or government?

YES NO

1. To which extent do you apply them (scale from 1 to 4)

- Not at all
- I follow some but not all
- I follow most of them
- I follow all the recommendations

1. How frequently do you touch your face?

- Never
- Rarely
- Sometimes
- Often
- Always

1. How do you feel regarding the current COVID-19 pandemic situation? (select all that applies)?

- Anxious/nervous
- Fear
- Angry
- Just fine
- Stressed Happy
- Lonely
- Bored
- Relaed/optimistic
- Having sleeping problems

1. How are you adapting? (select all that applies)

- Watching TV/movies
- Reading books/magazine
- Following Social media (Facebook/Whatsapp/Instagram)
- Volunteering
- Fighting with everyone around
- Work from home
- Spending time with family
- Practicing indoor sports
- Talking to myself
- Sleeping all the time
- Playing video games

1. On a scale of 1-5, how much stress/worry do you feel during the lock down / isolation?

1-Not stressed

5- Extremely stressed

**SECTION D: PERCEPTION Of GLOBAL RESPONSE**

1. Do you think that your government has/is doing enough to stop the global outbreak? Yes No I don’t know
2. Do you agree with the obligatory lockdown/measurements your country is taking? Yes No
3. Do you think that the WHO has/is doing enough to stop the global pandemic?

Yes NO

1. On a scale of 1-5, how satisfied are you with your country's response against the COVID-19 pandemic?
2. Not satisfied

5- Very satisfied

1. How satisfied are you with the media/social media coverage of the COVID-19 pandemic?

- Very satisfied/keeps me updated
- Makes me worry more/stressful
- Not enough information
- There are more lies than truth
- I don’t follow any media update
- No comment `

**SECTION E: COMMUNITY RESPONSE**

1. What do you think we can do as a community to reduce the spread of COVID-19?

- Follow/respect the Health recommendations of my country
- Eat Healthy/ Practice sports
- Social distancing/Avoid crowd
- Volunteer to support whenever possible
- Attending religious gatherings
- Avoid hand-shakes and face kissing
- I don’t know

1. Do you think we can prevent such global pandemics in the future?

Yes No I don’t Know

1. If yes, how?
   - - Reduce international travels
     - Improve surveillance in the human and animal health sectors
     - Establish early alerts and global warning systems for infectious diseases
     - Collaboration between environmental, animal, and human health workers
     - Intensify research on preventive measures such as vaccines/diagnosis
     - Raise public awareness of proper hygiene/healthy habits
     - Prioritize human life/health welfare over the animal or environmental ones
2. Are you willing to read and share with others information on COVID-19?

Yes NO

**Legends:**

*COVID-19* = Coronavirus Disease 2019

*TV* = Television
